# Hybrid feature-time series neural network for predicting ACL forces in martial artists with resistive braces after reconstruction

**DOI:** 10.3389/fbioe.2025.1579472

**Published:** 2025-05-09

**Authors:** Dongyue Li, Haojie Li, Yang Hang

**Affiliations:** ^1^ School of Physical Education, Guangzhou University, Guangzhou, China; ^2^ School of Exercise and Health, Shanghai University of Sport, Shanghai, China; ^3^ Physical Education Department, Guangdong University of Foreign Studies, Guangzhou, Guangdong, China

**Keywords:** ACL force prediction, hybrid neural network, temporal convolutional Network, resistive brace, postoperative rehabilitation

## Abstract

**Objective:**

This study developed a hybrid neural network integrating multi-modal data to predict anterior cruciate ligament (ACL) forces during rehabilitation in martial artists using a novel resistive knee brace after ACL reconstruction. The goal was to leverage time-series biomechanical parameters and static clinical features to optimize postoperative recovery strategies.

**Methods:**

A prospective cohort of 44 martial artists post-ACL reconstruction was randomized into an experimental group (EG, *n* = 22) using a resistive brace and a control group (CG, *n* = 22) using a traditional brace. Baseline demographics (height, weight), joint range of motion (ROM), and muscle strength were measured preoperatively (T0) and at 15 days (T1), 30 days (T2), and 60 days (T3) postoperatively. High-resolution kinematic and kinetic data were collected at T3, while ACL forces were computed at T3 using OpenSim musculoskeletal modeling. A feature-embedded temporal convolutional neural network (TCN) fused time-series gait data (T3) with static features (T0-T3) to predict ACL forces.

**Results:**

The hybrid TCN model achieved superior ACL force prediction accuracy, with a mean *R* ^2^ = 0.63 (EG), *R* ^2^ = 0.58 (CG), and *R* ^2^ = 0.62 (combined cohort) in three-fold cross-validation. Comparative analyses demonstrated significant advantages over standalone TCN (*R* ^2^ = 0.54) and long short-term memory (*R* ^2^ = 0.51) models.

**Conclusion:**

The integration of temporal biomechanical data and static clinical features enables accurate ACL force prediction, particularly for patients using resistive braces. This approach provides a novel tool to personalize rehabilitation protocols and validates the efficacy of resistive braces in modulating ACL loads, supporting their clinical adoption for athletes recovering from ACL injuries.

## 1 Introduction

After anterior cruciate ligament (ACL) reconstruction, the ligament mechanical environment is complex, and overloading may lead to graft failure, while underloading delays functional recovery (Musahl et al., 2022). ACL injuries are common injuries in high-intensity sports populations such as martial arts athletes, and the effectiveness of postoperative rehabilitation has a direct impact on the ability to return to sport (Dawkins et al., 2023). The key to postoperative rehabilitation after ACL reconstruction lies in the balancing of early functional training and ligament protection, but the Traditional rehabilitation programs rely on empirical adjustments and lack precise quantification of ligament forces, which may lead to overloading or inadequate rehabilitation (Garmasheva et al., 2021).

In recent years, resistance braces have been proposed to regulate joint loading through external resistance and theoretically reduce ACL stress by enhancing synergistic muscle contraction (Jalali et al., 2018). Studies have shown that resistance braces may improve gait symmetry (Focke et al., 2020) and reduce knee instability (Ewing et al., 2016), but the specific mechanism of their effect on ACL stress is unclear. Existing studies are mostly limited to mechanical simulation or short-term observation (Goerger et al., 2014), lacking the support of long-term dynamic monitoring data. For example, previous studies have found that functional braces can change knee kinematics, but have not quantified their effect on ACL loading (Palm et al., 2012); while another study pointed out that the biomechanical effects of different brace designs vary significantly, but lacked a predictive model to guide clinical selection (Rocchi et al., 2020). Therefore, an objective assessment method incorporating multimodal data is urgently needed to validate the mechanical efficacy of resistive braces and optimize their application. In addition, although the functional braces commonly used in clinical practice can limit the range of joint motion, they are unable to monitor the mechanical status of ACL in real time, resulting in a lack of individualized adjustment basis for the rehabilitation program (Mohseni et al., 2009). Therefore, the development of a method that can dynamically predict ACL forces is crucial for optimizing postoperative rehabilitation strategies.

Traditional ACL force prediction mainly relies on laboratory biomechanical models, which require high-precision motion capture and force table data, and is difficult to be applied to daily rehabilitation scenarios (Wu et al., 2001). While prediction models based on a single data source (e.g., LSTM dealing with gait time series) can capture dynamic features, they ignore the long-term effects of static clinical parameters (e.g., ROM, muscle strength) (Sinclair et al., 2019). In addition, static regression models (e.g., linear regression) cannot resolve the nonlinear relationship between gait dynamics and ACL forces (Valle et al., 2024). To address these issues, this study proposes a hybrid feature-time series neural network (TCN + static feature embedding) to achieve high-precision prediction of ACL force by fusing multimodal data (static features + dynamic gait) from preoperative to 60 days postoperatively.

The aim of this study was to develop a neural network model (TCN) combining static features and time-series data for the prediction of ligament forces in postoperative ACL patients using a resistive brace. The model innovatively integrates preoperative clinical data and postoperative gait characteristics to predict ACL loads in real time and guide the adjustment of rehabilitation programs. It provides data support for personalized rehabilitation. This study promotes the development of ACL postoperative rehabilitation in the direction of intelligence and precision.

## 2 Methods

### 2.1 Participants

This study recruited 44 professionally trained martial artists (age range: 18–30 years) following anterior cruciate ligament (ACL) reconstructive surgery as study participants.

#### 2.1.1 Inclusion criteria

1. Diagnosis of unilateral ACL insufficiency without concomitant intra-articular pathologies.
2. Surgical procedure: Anatomic ACL reconstruction utilizing autologous semitendinosus-gracilis double-bundle graft.
3. Demonstrated adherence to postoperative evaluation protocols and therapeutic regimens.
4. Restoration of functional knee mobility (0°–120° arc) with absence of mechanical locking or inflammatory effusion.

#### 2.1.2 Exclusion criteria

1. Complex knee trauma involving meniscal/capsuloligamentous structures.
2. Comorbid systemic conditions impairing neuromuscular performance (e.g., cerebrovascular accidents, class III/IV cardiac insufficiency).
3. Prior surgical interventions affecting bilateral knee joints.

To mitigate neural network training constraints associated with limited sample size, biomechanical parameters from 10 consecutive gait cycles per participant were systematically extracted, yielding 440 temporally resolved data streams for predictive modeling.

### 2.2 Study design

**FIGURE 1:**
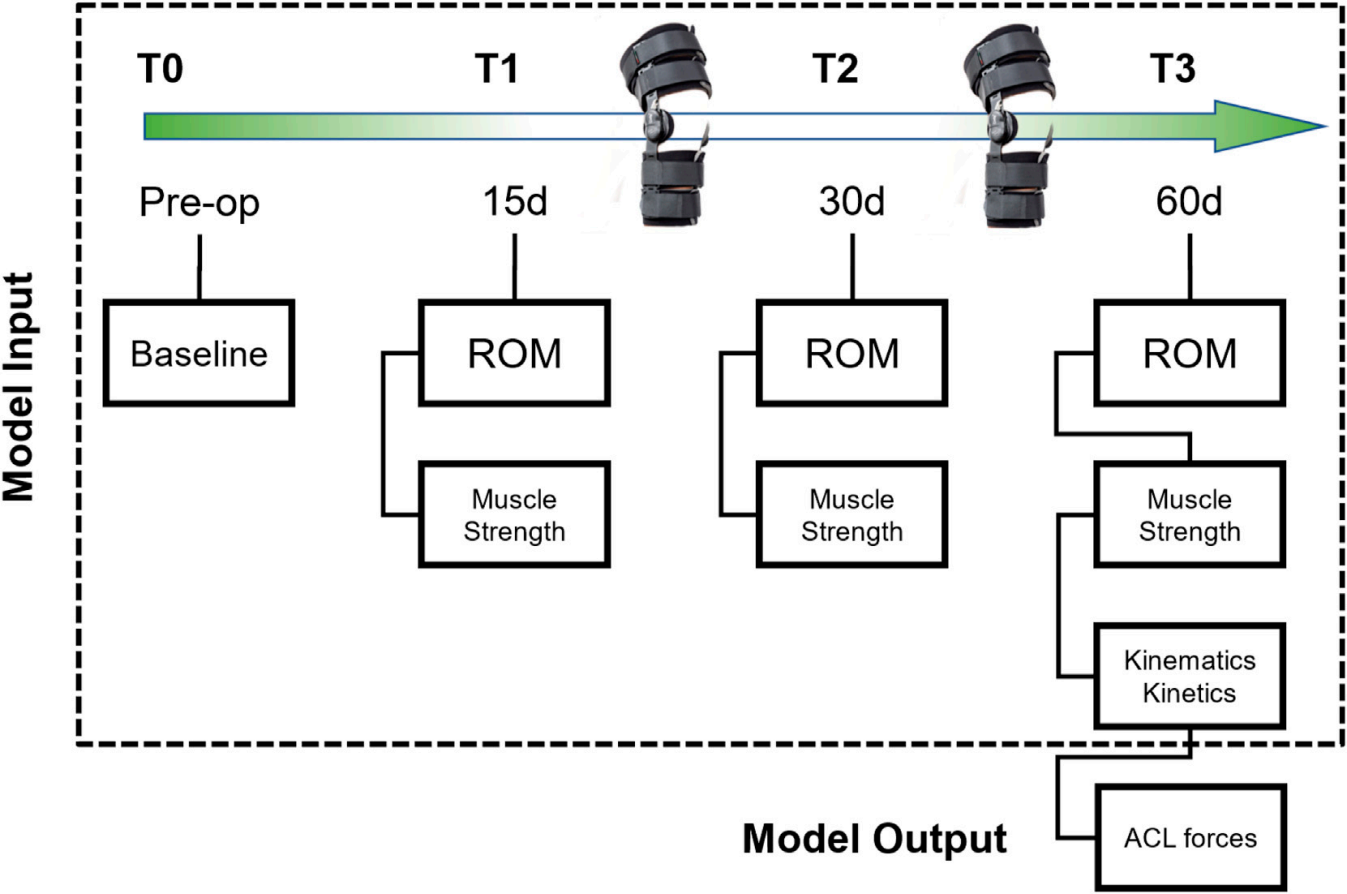
Study design flowchart.

This longitudinal cohort investigation investigated ACL force prediction using a hybrid neural network. Forty-four martial artists post-ACL reconstruction were randomly divided into two groups Figure 1.

• Experimental Group (EG, n = 22): Utilized a resistive knee brace (*RehaBrace, Italy*) providing 2.5 kg resistance. To establish a biomechanical dataset under resistance loading for training the neural network to predict ACL forces under active intervention.
• Control Group (CG, n = 22): Wore a traditional functional brace (*DA334-7, China*) with 0°–120° ROM. To validate model generalizability by predicting ACL forces under standard rehabilitation protocols without external resistance.

Both groups maintained brace-locked knee extension for the first 15 postoperative days, followed by progressive motion training.

#### 2.2.1 Intervention protocol

All participants received standardized rehabilitation.

1. Physical Therapy: 5 sessions/week (8 weeks) including passive mobilization and electrical stimulation.
2. Gait Training: 2–3 sessions/week (Weeks 3–8) on a treadmill at 1 m/s with interval pacing.

#### 2.2.2 Neural network inputs

Multi-source data were collected at four stages.

1. T0 (Pre-op): Baseline demographics (height, weight, BMI) and knee range of motion (ROM).
2. T1 (15 days): Muscle strength and ROM assessments.
3. T2 (30 days): Muscle strength and ROM assessments.
4. T3 (60 days): High-resolution kinematic and kinetic time-series data. ACL forces derived from musculoskeletal modeling as prediction labels.

The model integrated.

1. Static Features: T0-T2 variables (e.g., BMI, baseline ROM).
2. Time Series: T3 kinematics (hip/knee/angle trajectories) and kinetics (vertical GRF profiles).
3. Output: T3 normalized ACL force (BW).

### 2.3 Data collection and analysis

#### 2.3.1 Range of motion assessment

Knee flexion-extension angles during natural gait were measured using a high-precision electrogoniometer aligned with thigh/calf anatomical axes. Measurements were synchronized with force platform data (GRF threshold: 10 N for heel-strike detection).

#### 2.3.2 Muscle strength testing

Isokinetic quadriceps strength was assessed using a Biodex System Pro3 dynamometer (Biodex Corp., USA).

##### 2.3.2.1 Protocol

o *Warm-up*: 5 min submaximal exercise at 60°/s.
o *Testing*: Concentric peak torque measured at 30°/s, 60°/s (slow), 180°/s (medium), and 330°/s (high).
o *Positioning*: Seated with 70° hip flexion and 90° knee flexion, dynamometer axis aligned to knee joint.

##### 2.3.2.2 Execution

o Three maximal repetitions per speed with 5-min rest intervals.
o Randomized limb testing order with visual feedback.

#### 2.3.3 Motion capture

A three - dimensional motion capture system (qualisys system) was used to collect the kinematic data of the lower limb joints of the subjects during natural gait walking. The system is equipped with 8 infrared cameras, and the sampling frequency is 200 Hz. 48 reflective markers were precisely pasted on the skin of the subjects. The pasting positions include the left and right acromioclavicular joints, iliac crests, greater trochanters, medial and lateral epicondyles of the knee joint, medial and lateral malleoli, and the first and fifth metatarsal heads. In addition, there are the anterior superior iliac spine, posterior superior iliac spine, a rigid plate with four markers connected to the thoracic spine, bilateral thighs, and the lower legs with elastic Velcro straps, as well as three marker - rigid plates connected to the heels. The kinematic data reduction was calculated using Visual 3D software. The original kinematic and kinetic data were filtered using a fourth - order, zero - lag recursive Butterworth filter with a cut - off frequency of 20 Hz.

#### 2.3.4 ACL force computation

**FIGURE 2:**
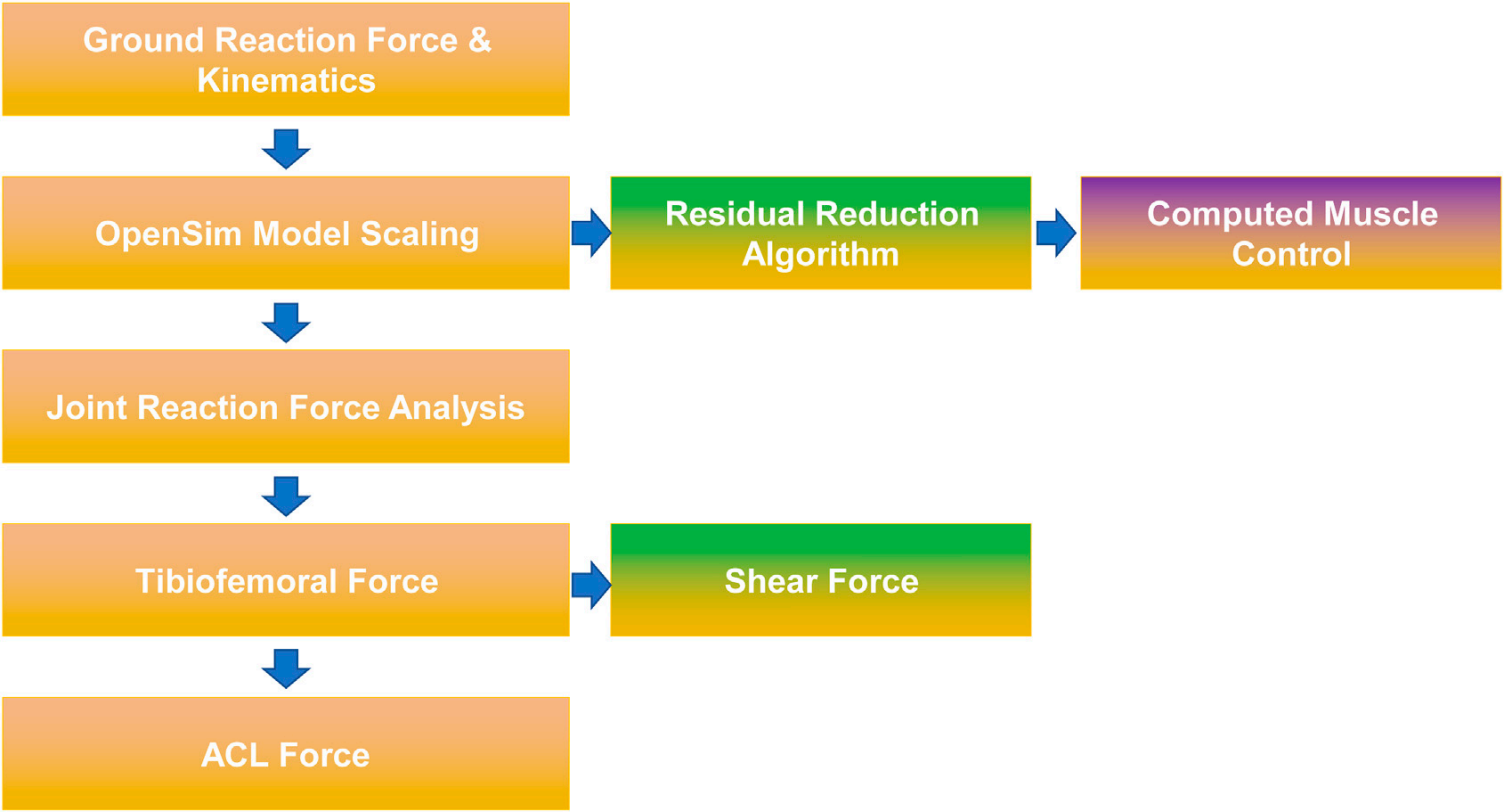
Acl force calculation schematic.

Ground reaction forces (1,000 Hz, Kistler platform) and motion data informed a scaled OpenSim musculoskeletal model (23 DOF, 92 muscles) Figure 2. Following residual reduction optimization, ACL forces were derived using the validated sagittal plane model by Kernozek and Ragan (Hachmann et al., 2021).

### 2.4 Neural network model construction

**FIGURE 3:**
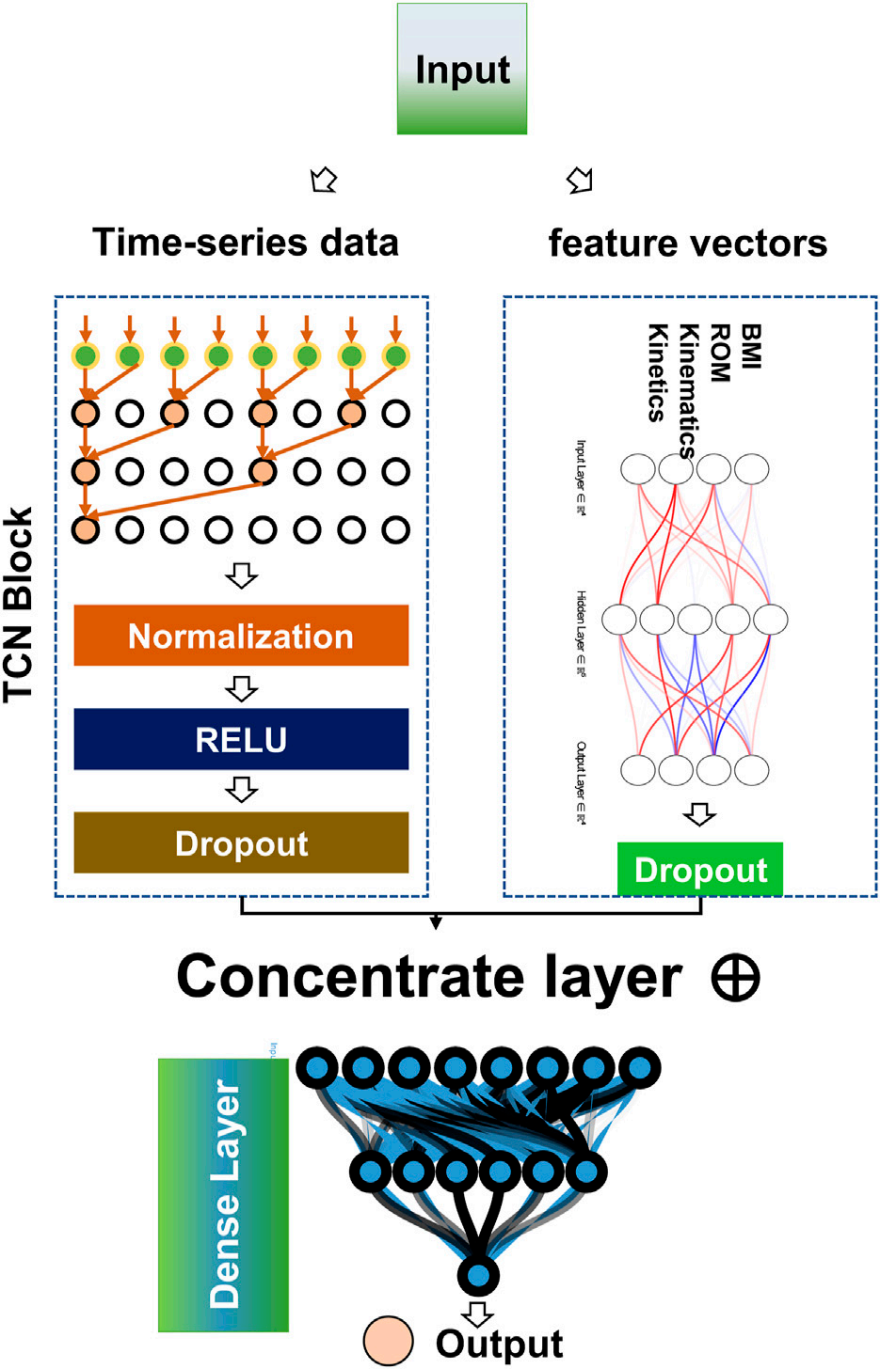
Architecture of neural networks.

This study constructed a hybrid neural network model for regression analysis using Python 3.8 and the Tensorflow framework Figure 3. The model consists of an input layer, hidden layers, and an output layer. The input layer receives two types of variables. Time-series data are fed into the model through a Temporal Convolutional Network (TCN). The processing of data in the TCN can be described by mathematical formulas. For the 1D convolutional layer, the operation formula is $y=\sigma \left(W*x+b\right)$ , where y is the output of the convolutional operation, σ is the activation function, W represents the weights of the convolutional kernel, * denotes the convolution operation, x is the input, and b is the bias term. In the TCN, data go through operations such as normalization, causal convolution, and dropout layers, and are processed using uniform TCN blocks before being transmitted to the concentrate layer.

The other type of variables, feature vectors, are processed by a fully connected layer with the formula $y=\sigma \left(Wx+b\right)$ and then input into the concentrate layer, where they are fused with the time-series data processed by the TCN. The fused data then pass through multiple fully connected layers and dropout layers, and finally, the output layer with only one neuron performs regression analysis and outputs the results.

The model training employs the Adam optimizer, which adaptively adjusts the learning rate for each parameter to accelerate model convergence. The update formulas of Adam optimizer are $m_{t}=\beta_{1}m_{t-1}+\left(1-\beta_{1}\right)g_{t}$ , $v_{t}=\beta_{2}v_{t-1}+\left(1-\beta_{2}\right)g_{t}^{2}$ , ${\hat{m}}_{t}=\frac{m_{t}}{1-\beta_{1}^{t}}$ , ${\hat{v}}_{t}=\frac{v_{t}}{1-\beta_{2}^{t}}$ , $\theta_{t+1}=\theta_{t}-\frac{\alpha {\hat{m}}_{t}}{\sqrt{{\hat{v}}_{t}}+\epsilon }$ , where $m_{t}$ and $v_{t}$ are the first-order and second-order moment estimates of the gradient respectively, $\beta_{1}$ and $\beta_{2}$ are the exponential decay rates of the moment estimates, $g_{t}$ is the current gradient, α is the learning rate, and ϵ is a small constant for numerical stability. Hyperparameters of the model, such as the learning rate, dilation rate of the convolutional layer, and dropout rate, are optimized using the grid search method. After determining the optimal combination of hyperparameters, the model will be trained for 1,000 epochs with a batch size of 32 each time.

### 2.5 Model validation

To evaluate the model performance more reliably, a three-fold cross-validation method is adopted. First, the preprocessed dataset is randomly divided into three non-overlapping subsets, denoted as A, B, and C. In the first round of validation, subset A is used as the test set, and subsets B and C are combined as the training set. The model is trained on the training set and then evaluated on the test set A, and evaluation metrics such as $R^{2}$ and RMSE are recorded. The formulas for $R^{2}$ and RMSE are $R^{2}=1-\frac{\sum_{i=1}^{n}\;{\left(y_{i}-{\hat{y}}_{i}\right)}^{2}}{\sum_{i=1}^{n}\;{\left(y_{i}-\bar{y}\right)}^{2}}$ and $RMSE=\sqrt{\frac{1}{n}\sum_{i=1}^{n}\;{\left(y_{i}-{\hat{y}}_{i}\right)}^{2}}$ , where n is the number of samples, $y_{i}$ is the true value, ${\hat{y}}_{i}$ is the predicted value, and $\bar{y}$ is the mean of the true values. Then, in the second round of validation, subset B is used as the test set, and subsets A and C form the training set, repeating the training and evaluation process. Finally, in the third round of validation, subset C is used as the test set, and subsets A and B are used as the training set for training and evaluation again. After completing the three rounds of validation, the average values of $R^{2}$ and RMSE obtained from the three rounds are calculated as the final performance evaluation results of the model. This method effectively avoids the influence of specific data divisions on the model performance and ensures the robustness and reliability of the evaluation results. To benchmark predictive efficacy, the proposed model was rigorously compared against standalone TCN and Long Short-Term Memory (LSTM) architectures.

## 3 Results

Demographic details are presented in Table 1.

**TABLE 1:** Demographic information of participants.

	EG	CG
Gender	50% female	45% female
Age (years)	24.3 ± 3.2	25.3 ± 3.9
Height (cm)	171.3 ± 7.4	172.3 ± 7.7
Weight (kg)	66.5 ± 8.7	65.5 ± 9.1
Training Experience (years)	13.7 ± 4.6	14.2 ± 5.7
Knee range of motion (°)		
T0	45.6 ± 6.9	43.2 ± 6.2
T1	13.2 ± 8.8	16.1 ± 9.0
T2	34.4 ± 6.6	25.4 ± 7.4
T3	47.1 ± 7.3	37.7 ± 8.8
F_ACL_	0.51 ± 0.13	0.63 ± 0.17

Note: The ACL, Force (F_ACL_) was standardized according to the body weight (BW) of the subjects.

**FIGURE 4:**
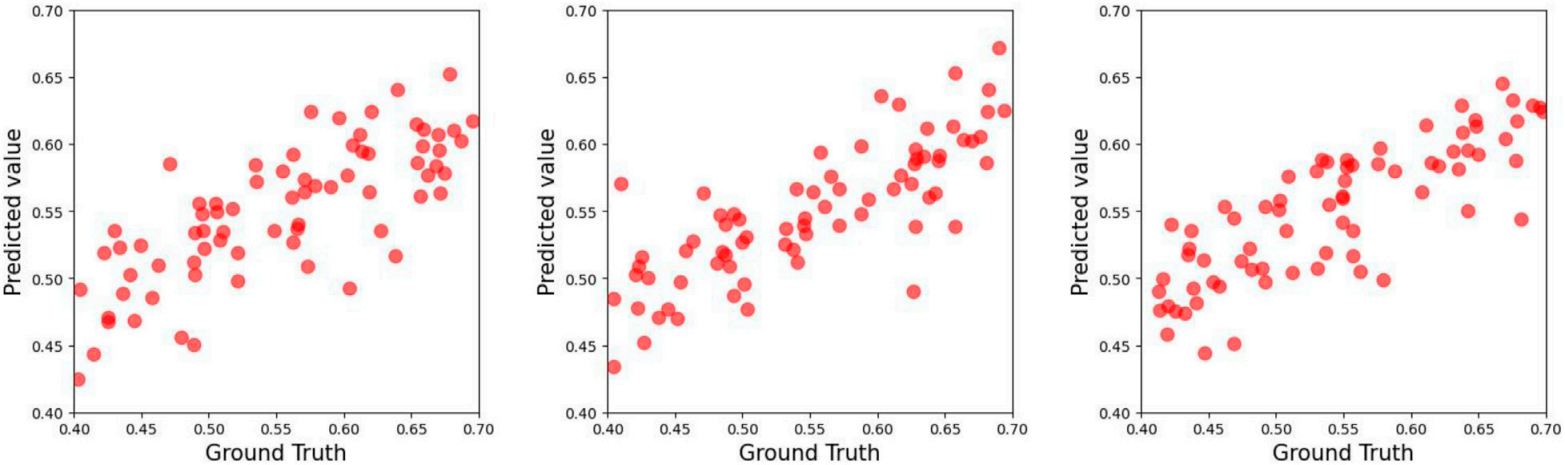
Scatter plot of the test set of the experimental group in the three-fold cross-validation for predicting ACL forces.

**FIGURE 5:**
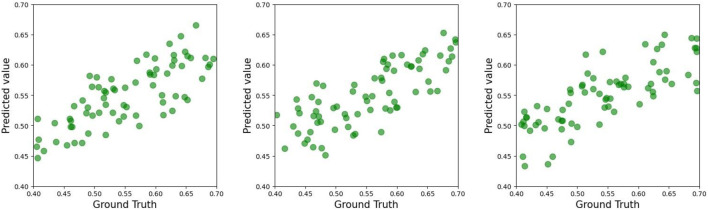
Scatter plot of the test set of the control group in the three-fold cross-validation for predicting ACL forces.

**FIGURE 6:**
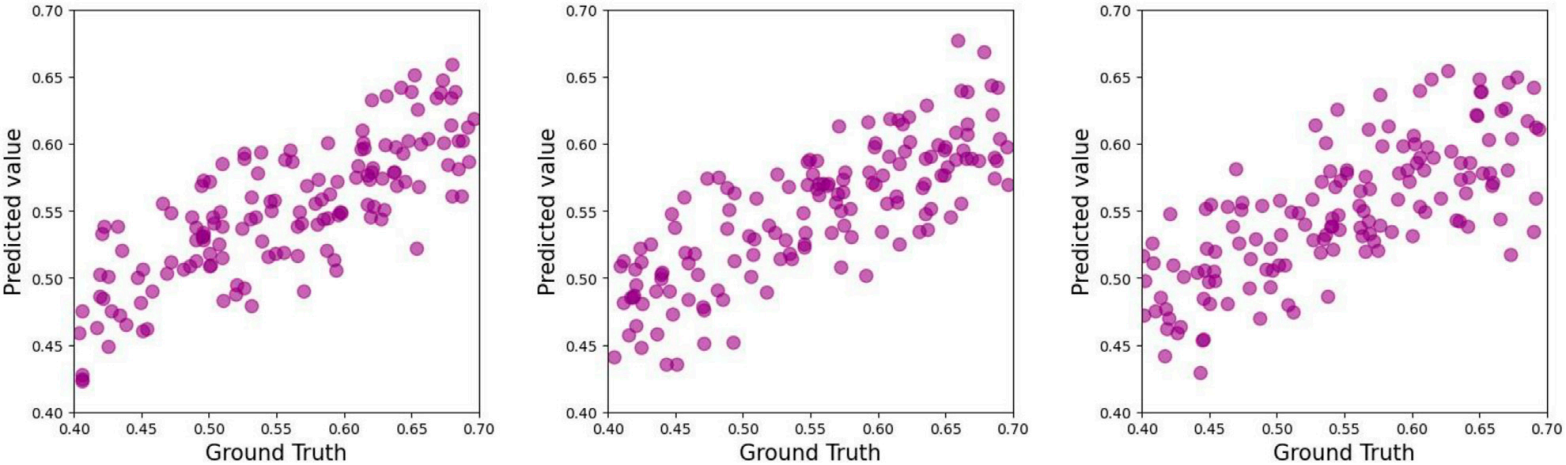
Scatter plot of the test set of all groups in the three-fold cross-validation for predicting ACL forces.

As demonstrated in Tables 2–4, the **TCN-based hybrid model incorporating feature embeddings** achieved robust predictive accuracy for ACL force estimation across the experimental group, control group, and combined cohort. Notably, the experimental group exhibited the highest performance, with a mean coefficient of determination (*R* ^2^) of 0.63 in three-fold cross-validation (see Figures 4–6).

**TABLE 2:** Results of Three - fold Cross - Validation for Predicting ACL Forces in the Experimental Group.

	Train *R* ^2^	Test *R* ^2^	Average test *R* ^2^
Fold 1	0.77	0.61	0.63
Fold 2	0.75	0.65	
Fold 3	0.76	0.64	

**TABLE 3:** Results of Three - fold Cross - Validation for Predicting ACL Forces in the Control Group.

	Train *R* ^2^	Test *R* ^2^	Average test *R* ^2^
Fold 1	0.61	0.56	0.58
Fold 2	0.62	0.61	
Fold 3	0.66	0.59	

**TABLE 4:** Results of Three - fold Cross - Validation for Predicting ACL Forces in All Group.

	Train *R* ^2^	Test *R* ^2^	Average test *R* ^2^
Fold 1	0.71	0.61	0.62
Fold 2	0.65	0.64	
Fold 3	0.72	0.61	

**FIGURE 7:**
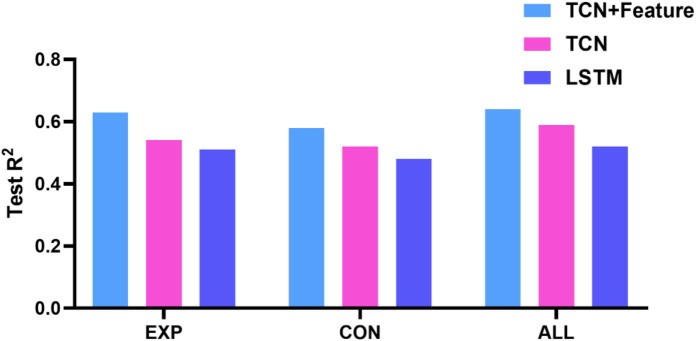
Mean value of R-squared for cross-validation of different models.

Comparative analyses (Figure 7) revealed that the **TCN + feature embedding architecture** demonstrated superior predictive accuracy compared to standalone TCN (*R* ^2^ = 0.54) and LSTM (*R* ^2^ = 0.51) models, underscoring the efficacy of multimodal feature fusion in biomechanical force modeling.

## 4 Discussion

The hybrid TCN model developed in this study showed a significant advantage in ACL force prediction (*R* ^2^ = 0.63 for the experimental group), a result that outperforms traditional biomechanical models and single-modal prediction methods. In contrast to medical diagnostic neural networks proposed in previous studies (Kernozek and Ragan, 2008), our model innovatively fuses static clinical features with dynamic gait data, which is consistent with the theoretical findings of previous studies on multimodal neural networks (Wang et al., 2023). However, compared with previous studies that used only kinetic data to predict ACL loads, our method significantly improved in prediction accuracy (Shi et al., 2022), which validates the important contribution of static features to long-term prediction. Notably, our results are consistent with findings regarding the importance of temporal features (Cordero-Sánchez et al., 2025), but confirm for the first time that the synergistic effect of static and dynamic features is even more critical under resistance brace conditions. This study is of great value for the postoperative rehabilitation of wushu athletes. By accurately predicting the changes in ACL forces, it can help clinicians to develop individualized rehabilitation programs to avoid secondary ligament injuries during training (Zangene et al., 2021); meanwhile, athletes can adjust their training intensity and movement techniques according to the prediction results to safely and efficiently regain their athletic abilities (Wilhelm et al., 2024).

In addition, this study showed that the resistance brace significantly improved ROM recovery (47.1° vs. 37.7°) and reduced ACL loading (0.51 B W vs. 0.63 B W), a finding that is consistent with previous studies (Palmieri-Smith et al., 2022), but this study is the first to quantify the mechanical mechanism through neural network modeling. Compared with the previously reported effects of conventional braces, the resistance design showed unique advantages in improving joint coordination (Wu et al., 2023). The study provides a new basis for the optimal design of resistance braces. These findings provide important guidance for the rehabilitation training of wushu athletes: through real-time monitoring of ACL force changes, athletes can adjust their training intensity in a timely manner (Dutton et al., 2014), and gradually resume difficult movements under the premise of ensuring safety; and clinicians can formulate individualized brace use plans for each athlete based on the prediction results to optimize the rehabilitation process (Hiller and Beckenkamp, 2023).

The real-time prediction ability (*R* ^2^ > 0.6) of this study is significantly better than that of the traditional OpenSim modeling approach, and our scheme extends the clinical applicability significantly (from the laboratory environment to routine rehabilitation scenarios) compared to earlier studies (Reis et al., 2024). Although previous studies have proposed similar concepts for personalized rehabilitation (Chidambaram et al., 2022), this study is the first to achieve quantitative decision support based on neural networks. Accurate ACL force data can be obtained through a simple gait analysis device to develop a more scientific rehabilitation program for athletes; at the same time, athletes can understand the ligament loading during training in real time, and adjust the amplitude and intensity of movements in a timely manner, which ensures the rehabilitation effect as well as avoids secondary injuries (Duhig and McKenzie, 2024).

Limitations of the study: this study has certain limitations. Although the sample size meets the basic statistical requirements, it may affect the applicability of the model in a wider population. In addition, the data collection relied on laboratory equipment, which has some limitations in clinical application. These factors need to be further optimized in future studies.

## 5 Conclusion

The hybrid TCN model developed in this study successfully achieved high-precision prediction of ACL forces (*R* ^2^ = 0.63 for the experimental group), confirming the key role of integrating dynamic gait data with static clinical characteristics to enhance prediction. The resistance brace significantly improved knee mobility and reduced ACL loading, and its biomechanical benefits were quantitatively validated by neural network modeling for the first time. The predictive model breaks through the limitations of traditional laboratory methods and provides a practical and personalized rehabilitation decision-making tool for clinical practice, which supports the promotion of the use of resistive braces in post-operative ACL rehabilitation. Future studies need to expand the sample size and optimize the data collection method to further enhance the generalizability of the model.

## Data Availability

The original contributions presented in the study are included in the article/supplementary material, further inquiries can be directed to the corresponding author.
